# Students' Achievement and Homework Assignment Strategies

**DOI:** 10.3389/fpsyg.2017.00286

**Published:** 2017-03-07

**Authors:** Rubén Fernández-Alonso, Marcos Álvarez-Díaz, Javier Suárez-Álvarez, José Muñiz

**Affiliations:** ^1^Department of Education Sciences, University of OviedoOviedo, Spain; ^2^Department of Education, Principality of Asturias GovernmentOviedo, Spain; ^3^Department of Psychology, University of OviedoOviedo, Spain

**Keywords:** homework time, equity, compulsory secondary education, hierarchical modeling, adolescents

## Abstract

The optimum time students should spend on homework has been widely researched although the results are far from unanimous. The main objective of this research is to analyze how homework assignment strategies in schools affect students' academic performance and the differences in students' time spent on homework. Participants were a representative sample of Spanish adolescents (*N* = 26,543) with a mean age of 14.4 (±0.75), 49.7% girls. A test battery was used to measure academic performance in four subjects: Spanish, Mathematics, Science, and Citizenship. A questionnaire allowed the measurement of the indicators used for the description of homework and control variables. Two three-level hierarchical-linear models (student, school, autonomous community) were produced for each subject being evaluated. The relationship between academic results and homework time is negative at the individual level but positive at school level. An increase in the amount of homework a school assigns is associated with an increase in the differences in student time spent on homework. An optimum amount of homework is proposed which schools should assign to maximize gains in achievement for students overall.

The role of homework in academic achievement is an age-old debate (Walberg et al., 1985) that has swung between times when it was thought to be a tool for improving a country's competitiveness and times when it was almost outlawed. So Cooper (2001) talks about the *battle over homework* and the debates and rows continue (Walberg et al., 1985, 1986; Barber, 1986). It is considered a *complicated subject* (Corno, 1996), *mysterious* (Trautwein and Köller, 2003), *a chameleon* (Trautwein et al., 2009b), or *Janus-faced* (Flunger et al., 2015). One must agree with Cooper et al. (2006) that homework is a practice full of contradictions, where positive and negative effects coincide. As such, depending on our preferences, it is possible to find data which support the argument that homework benefits all students (Cooper, 1989), or that it does not matter and should be abolished (Barber, 1986). Equally, one might argue a compensatory effect as it favors students with more difficulties (Epstein and Van Voorhis, 2001), or on the contrary, that it is a source of inequality as it specifically benefits those better placed on the social ladder (Rømming, 2011). Furthermore, this issue has jumped over the school wall and entered the home, contributing to the polemic by becoming a common topic about which it is possible to have an opinion without being well informed, something that Goldstein (1960) warned of decades ago after reviewing almost 300 pieces of writing on the topic in *Education Index* and finding that only 6% were empirical studies.

The relationship between homework time and educational outcomes has traditionally been the most researched aspect (Cooper, 1989; Cooper et al., 2006; Fan et al., 2017), although conclusions have evolved over time. The first experimental studies (Paschal et al., 1984) worked from the hypothesis that time spent on homework was a reflection of an individual student's commitment and diligence and as such the relationship between time spent on homework and achievement should be positive. This was roughly the idea at the end of the twentieth century, when more positive effects had been found than negative (Cooper, 1989), although it was also known that the relationship was not strictly linear (Cooper and Valentine, 2001), and that its strength depended on the student's age- stronger in post-compulsory secondary education than in compulsory education and almost zero in primary education (Cooper et al., 2012). With the turn of the century, hierarchical-linear models ran counter to this idea by showing that homework was a multilevel situation and the effect of homework on outcomes depended on classroom factors (e.g., frequency or amount of assigned homework) more than on an individual's attitude (Trautwein and Köller, 2003). Research with a multilevel approach indicated that individual variations in time spent had little effect on academic results (Farrow et al., 1999; De Jong et al., 2000; Dettmers et al., 2010; Murillo and Martínez-Garrido, 2013; Fernández-Alonso et al., 2014; Núñez et al., 2014; Servicio de Evaluación Educativa del Principado de Asturias, 2016) and that when statistically significant results were found, the effect was negative (Trautwein, 2007; Trautwein et al., 2009b; Lubbers et al., 2010; Chang et al., 2014). The reasons for this null or negative relationship lie in the fact that those variables which are positively associated with homework time are antagonistic when predicting academic performance. For example, some students may not need to spend much time on homework because they learn quickly and have good cognitive skills and previous knowledge (Trautwein, 2007; Dettmers et al., 2010), or maybe because they are not very persistent in their work and do not finish homework tasks (Flunger et al., 2015). Similarly, students may spend more time on homework because they have difficulties learning and concentrating, low expectations and motivation or because they need more direct help (Trautwein et al., 2006), or maybe because they put in a lot of effort and take a lot of care with their work (Flunger et al., 2015). Something similar happens with sociological variables such as gender: Girls spend more time on homework (Gershenson and Holt, 2015) but, compared to boys, in standardized tests they have better results in reading and worse results in Science and Mathematics (OECD, 2013a).

On the other hand, thanks to multilevel studies, systematic effects on performance have been found when homework time is considered at the class or school level. De Jong et al. (2000) found that the number of assigned homework tasks in a year was positively and significantly related to results in mathematics. Equally, the volume or amount of homework (mean homework time for the group) and the frequency of homework assignment have positive effects on achievement. The data suggests that when frequency and volume are considered together, the former has more impact on results than the latter (Trautwein et al., 2002; Trautwein, 2007). In fact, it has been estimated that in classrooms where homework is always assigned there are gains in mathematics and science of 20% of a standard deviation over those classrooms which sometimes assign homework (Fernández-Alonso et al., 2015). Significant results have also been found in research which considered only homework volume at the classroom or school level. Dettmers et al. (2009) concluded that the school-level effect of homework is positive in the majority of participating countries in PISA 2003, and the OECD (2013b), with data from PISA 2012, confirms that schools in which students have more weekly homework demonstrate better results once certain school and student-background variables are discounted. To put it briefly, homework has a multilevel nature (Trautwein and Köller, 2003) in which the variables have different significance and effects according to the level of analysis, in this case a positive effect at class level, and a negative or null effect in most cases at the level of the individual. Furthermore, the fact that the clearest effects are seen at the classroom and school level highlights the role of homework policy in schools and teaching, over and above the time individual students spend on homework.

From this complex context, this current study aims to explore the relationships between the strategies schools use to assign homework and the consequences that has on students' academic performance and on the students' own homework strategies. There are two specific objectives, firstly, to systematically analyze the differential effect of time spent on homework on educational performance, both at school and individual level. We hypothesize a positive effect for homework time at school level, and a negative effect at the individual level. Secondly, the influence of homework quantity assigned by schools on the distribution of time spent by students on homework will be investigated. This will test the previously unexplored hypothesis that an increase in the amount of homework assigned by each school will create an increase in differences, both in time spent on homework by the students, and in academic results. Confirming this hypothesis would mean that an excessive amount of homework assigned by schools would penalize those students who for various reasons (pace of work, gaps in learning, difficulties concentrating, overexertion) need to spend more time completing their homework than their peers. In order to resolve this apparent paradox we will calculate the optimum volume of homework that schools should assign in order to benefit the largest number of students without contributing to an increase in differences, that is, without harming educational equity.

## Methods

### Participants

The population was defined as those students in year 8 of compulsory education in the academic year 2009/10 in Spain. In order to provide a representative sample, a stratified random sampling was carried out from the 19 autonomous regions in Spain. The sample was selected from each stratum according to a two-stage cluster design (OECD, 2009, 2011, 2014a; Ministerio de Educación, 2011). In the first stage, the primary units of the sample were the schools, which were selected with a probability proportional to the number of students in the 8th grade. The more 8th grade students in a given school, the higher the likelihood of the school being selected. In the second stage, 35 students were selected from each school through simple, systematic sampling. A detailed, step-by-step description of the sampling procedure may be found in OECD (2011). The subsequent sample numbered 29,153 students from 933 schools. Some students were excluded due to lack of information (absences on the test day), or for having special educational needs. The baseline sample was finally made up of 26,543 students. The mean student age was 14.4 with a standard deviation of 0.75, rank of age from 13 to 16. Some 66.2% attended a state school; 49.7% were girls; 87.8% were Spanish nationals; 73.5% were in the school year appropriate to their age, the remaining 26.5% were at least 1 year behind in terms of their age.

### Procedure

Test application, marking, and data recording were contracted out via public tendering, and were carried out by qualified personnel unconnected to the schools. The evaluation, was performed on two consecutive days, each day having two 50 min sessions separated by a break. At the end of the second day the students completed a context questionnaire which included questions related to homework. The evaluation was carried out in compliance with current ethical standards in Spain. Families of the students selected to participate in the evaluation were informed about the study by the school administrations, and were able to choose whether those students would participate in the study or not.

### Instruments

#### Tests of academic performance

The performance test battery consisted of 342 items evaluating four subjects: Spanish (106 items), mathematics (73 items), science (78), and citizenship (85). The items, completed on paper, were in various formats and were subject to binary scoring, except 21 items which were coded on a polytomous scale, between 0 and 2 points (Ministerio de Educación, 2011). As a single student is not capable of answering the complete item pool in the time given, the items were distributed across various booklets following a matrix design (Fernández-Alonso and Muñiz, 2011). The mean Cronbach α for the booklets ranged from 0.72 (mathematics) to 0.89 (Spanish). Student scores were calculated adjusting the bank of items to Rasch's IRT model using the ConQuest 2.0 program (Wu et al., 2007) and were expressed in a scale with mean and standard deviation of 500 and 100 points respectively. The student's scores were divided into five categories, estimated using the plausible values method. In large scale assessments this method is better at recovering the true population parameters (e.g., mean, standard deviation) than estimates of scores using methods of maximum likelihood or expected a-posteriori estimations (Mislevy et al., 1992; OECD, 2009; von Davier et al., 2009).

### Homework variables

A questionnaire was made up of a mix of items which allowed the calculation of the indicators used for the description of homework variables. *Daily minutes spent on homework* was calculated from a multiple choice question with the following options: (a) Generally I don't have homework; (b) 1 h or less; (c) Between 1 and 2 h; (d) Between 2 and 3 h; (e) More than 3 h. The options were recoded as follows: (a) = 0 min.; (b) = 45 min.; (c) = 90 min.; (d) = 150 min.; (e) = 210 min. According to Trautwein and Köller (2003) the average homework time of the students in a school could be regarded as a good proxy for the amount of homework assigned by the teacher. So the mean of this variable for each school was used as an estimator of *Amount or volume of homework assigned*.

### Control variables

Four variables were included to describe sociological factors about the students, three were binary: *Gender* (1 = *female*); *Nationality* (1 = *Spanish; 0* = *other*); School type (1 = *state school; 0* = *private*). The fourth variable was *Socioeconomic and cultural index* (SECI), which is constructed with information about family qualifications and professions, along with the availability of various material and cultural resources at home. It is expressed in standardized points, *N(0,1)*. Three variables were used to gather educational history: *Appropriate School Year* (1 = *being in the school year appropriate to their age*; 0 = *repeated a school year)*. The other two adjustment variables were *Academic Expectations* and *Motivation* which were included for two reasons: they are both closely connected to academic achievement (Suárez-Álvarez et al., 2014). Their position as adjustment factors is justified because, in an ex-post facto descriptive design such as this, both expectations and motivation may be thought of as background variables that the student brings with them on the day of the test. *Academic expectations for finishing education* was measured with a multiple-choice item where the score corresponds to the years spent in education in order to reach that level of qualification: compulsory secondary education (10 points); further secondary education (12 points); non-university higher education (14 points); University qualification (16 points). *Motivation* was constructed from the answers to six four-point Likert items, where 1 means strongly disagree with the sentence and 4 means strongly agree. Students scoring highly in this variable are agreeing with statements such as “at school I learn useful and interesting things.” A Confirmatory Factor Analysis was performed using a Maximum Likelihood robust estimation method (MLMV) and the items fit an essentially unidimensional scale: CFI = 0.954; TLI = 0.915; SRMR = 0.037; RMSEA = 0.087 (90% CI = 0.084–0.091).

As this was an official evaluation, the tests used were created by experts in the various fields, contracted by the Spanish Ministry of Education in collaboration with the regional education authorities.

### Data analyses

Firstly the descriptive statistics and Pearson correlations between the variables were calculated. Then, using the HLM 6.03 program (Raudenbush et al., 2004), two three-level hierarchical-linear models (student, school, autonomous community) were produced for each subject being evaluated: a null model (without predictor variables) and a random intercept model in which adjustment variables and homework variables were introduced at the same time. Given that HLM does not return standardized coefficients, all of the variables were standardized around the general mean, which allows the interpretation of the results as classical standardized regression analysis coefficients. Levels 2 and 3 variables were constructed from means of standardized level 1 variables and were not re-standardized. Level 1 variables were introduced without centering except for four cases: study time, motivation, expectation, and socioeconomic and cultural level which were centered on the school mean to control composition effects (Xu and Wu, 2013) and estimate the effect of differences in homework time among the students within the same school. The range of missing variable cases was very small, between 1 and 3%. Recovery was carried out using the procedure described in Fernández-Alonso et al. (2012).

The results are presented in two ways: the tables show standardized coefficients while in the figures the data are presented in a real scale, taking advantage of the fact that a scale with a 100 point standard deviation allows the expression of the effect of the variables and the differences between groups as percentage increases in standardized points.

## Results

Table 1 shows the descriptive statistics and the matrix of correlations between the study variables. As can be seen in the table, the relationship between the variables turned out to be in the expected direction, with the closest correlations between the different academic performance scores and socioeconomic level, appropriate school year, and student expectations. The nationality variable gave the highest asymmetry and kurtosis, which was to be expected as the majority of the sample are Spanish.

**Table 1:** **Descriptive statistics and Pearson correlation matrix between the variables**.

	**1**	**2**	**3**	**4**	**5**	**6**	**7**	**8**	**9**	**10**	**11**	**12**	**13**	**14**	**15**
1. Mathematics	–														
2. Spanish	0.45	–													
3. Sciences	0.48	0.61	–												
4. Citizenship	0.42	0.59	0.55	–											
5. SEC	0.29	0.36	0.34	0.29	–										
6. Female	−0.05	0.11	−0.05	0.13	−0.01	–									
7. Spanish national	0.12	0.16	0.14	0.12	0.18	−0.01	–								
8. Appropriate school year	0.26	0.34	0.32	0.28	0.31	0.08	0.15	–							
9. Expectations	0.26	0.38	0.33	0.35	0.36	0.13	0.07	0.42	–						
10. Motivation	0.02	0.06	0.06	0.11	−0.02	0.12	−0.04	0.06	0.16	–					
11. Homework time	0.03	0.07	0.05	0.07	0.13	0.14	0.02	0.14	0.19	0.16	–				
12. State school	−0.15	−0.21	−0.17	−0.19	−0.29	−0.01	−0.09	−0.12	−0.16	−0.01	−0.09	–			
13.School SEC	0.25	0.31	0.28	0.24	0.55	0.01	0.11	0.21	0.23	−0.06	0.09	−0.53	–		
14. HWTIME_mean	0.09	0.12	0.11	0.13	0.15	0.04	0.08	0.06	0.11	0.07	0.34	−0.26	0.27	–	
15. AC SEC	0.17	0.16	0.16	0.11	0.24	0.01	−0.04	0.10	0.05	−0.13	−0.04	−0.17	0.44	−0.10	–
Mean	506.47	509.65	509.37	508.10	0.06	0.50	0.88	0.74	14.06	2.87	91.26	0.66	0.06	91.26	0.06
Standard deviation	99.44	95.69	96.37	97.08	1,00	0.50	0.33	0.43	2,34	0.49	42.40	0.48	0.55	14.35	0.24
Asymmetry	0.17	−0.14	−0.05	−0.18	−0.18	−0.03	−2.34	−1.19	−0.54	−0.39	1.26	−0.65	0.01	0.67	−0.11
Kurtosis	0.13	0.11	0.05	−0.07	−0.53	−2.00	3.46	−0.59	−1.48	0.62	1.87	−1.58	−0.01	1.20	−0.55

Table 2 shows the distribution of variance in the null model. In the four subjects taken together, 85% of the variance was found at the student level, 10% was variance between schools, and 5% variance between regions. Although the 10% of variance between schools could seem modest, underlying that there were large differences. For example, in Spanish the 95% plausible value range for the school means ranged between 577 and 439 points, practically 1.5 standard deviations, which shows that schools have a significant impact on student results.

**Table 2:** **Distribution of the variance in the null model**.

**Variance**	**Mathematics**	**Sciences**	**Spanish**	**Citizenship**
Level 1	0.8754	0.8521	0.8191	0.8391
Level 2	0.0771	0.1048	0.1353	0.1259
Level 3	0.0482	0.0508	0.0572	0.0430

Table 3 gives the standardized coefficients of the independent variables of the four multilevel models, as well as the percentage of variance explained by each level.

**Table 3:** **Multilevel models for prediction of achievement in four subjects**.

	**Mathematics**	**Sciences**	**Spanish**	**Citizenship**
	**β (SE)**	**β (SE)**	**β (SE)**	**β (SE)**
**CONTROL VARIABLES**				
**Level 1 (student)**				
SECI	0.126 (0.010)^***^	0.144 (0.008)^***^	0.151 (0.009)^***^	0.116 (0.007)^***^
Women	−0.072 (0.007)^***^	−0.089 (0.007)^***^	0.068 (0.007)^***^	0.089 (0.008)^***^
Country: Spain	0.060 (0.008)^***^	0.069 (0.008)^***^	0.088 (0.007)^***^	0.060 (0.007)^***^
Appropriate school year	0.129 (0.008)^***^	0.162 (0.008)^***^	0.158 (0.008)^***^	0.127 (0.007)^***^
Expectations	0.146 (0.009)^***^	0.191 (0.011)^***^	0.211 (0.008)^***^	0.204 (0.007)^***^
Motivation	0.026 (0.007)^**^	0.058 (0.008)^***^	0.035 (0.006)^***^	0.066 (0.007)^***^
**Level 2 (school)**				
State school	−0.021 (0.014)	−0.027 (0.012)^*^	−0.054 (0.013)^***^	−0.077 (0.013)^***^
School SECI	0.163 (0.013)^***^	0.177 (0.013)^***^	0.192 (0.020)^***^	0.132 (0.013)^***^
**Level 3 (AC)**				
AC SECI	0.370 (0.123)^**^	0.261 (0.247)	0.224 (0.225)	0.131 (0.237)
**HW Variables**				
HW Time (student)	−0.050 (0.008)^***^	−0.053 (0.006)^***^	−0.055 (0.006)^***^	−0.055 (0.007)^***^
HW Amount (school)	0.046 (0.011)^***^	0.075 (0.009)^***^	0.068 (0.011)^***^	0.083 (0.011)^***^
**Percentage of variance explained**				
Level 1	9.7	15.9	18.7	15.0
Level 2	57.1	58.7	59.3	47.7
Level 3	67.3	53.0	50.1	36.2
Total	16.1	22.2	25.9	20.0

* *p < 0.05*;

** p < 0.01;

*** *p < 0.001*.

*β, Standardized weight; SE, Standard Error; SECI, Socioeconomic and cultural index; AC, Autonomous Communities*.

The results indicated that the adjustment variables behaved satisfactorily, with enough control to analyze the net effects of the homework variables. This was backed up by two results, firstly, the two variables with highest standardized coefficients were those related to educational history: academic expectations at the time of the test, and being in the school year corresponding to age. Motivation demonstrated a smaller effect but one which was significant in all cases. Secondly, the adjustment variables explained the majority of the variance in the results. The percentages of total explained variance in Table 2 were calculated with all variables. However, if the strategy had been to introduce the adjustment variables first and then add in the homework variables, the explanatory gain in the second model would have been about 2% in each subject.

The amount of homework turned out to be positively and significantly associated with the results in the four subjects. In a 100 point scale of standard deviation, controlling for other variables, it was estimated that for each 10 min added to the daily volume of homework, schools would achieve between 4.1 and 4.8 points more in each subject, with the exception of mathematics where the increase would be around 2.5 points. In other words, an increase of between 15 and 29 points in the school mean is predicted for each additional hour of homework volume of the school as a whole. This school level gain, however, would only occur if the students spent exactly the same time on homework as their school mean. As the regression coefficient of student homework time is negative and the variable is centered on the level of the school, the model predicts deterioration in results for those students who spend more time than their class mean on homework, and an improvement for those who finish their homework more quickly than the mean of their classmates.

**Figure 1:**
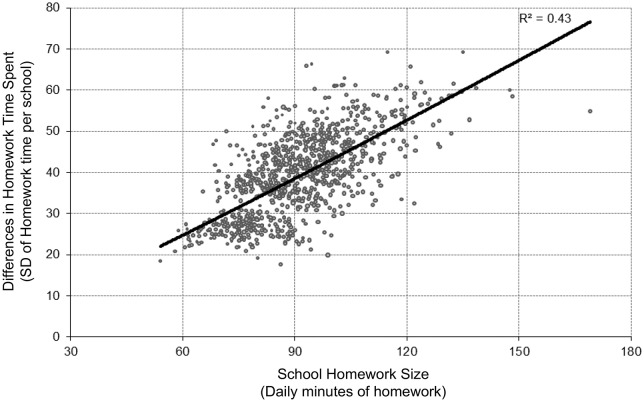
**Relationship between school homework volume and differences in time needed by students to complete homework**.

Furthermore, the results demonstrated a positive association between the amount of homework assigned in a school and the differences in time needed by the students to complete their homework. Figure 1 shows the relationship between volume of homework (expressed as mean daily minutes of homework by school) and the differences in time spent by students (expressed as the standard deviation from the mean school daily minutes). The correlation between the variables was 0.69 and the regression gradient indicates that schools which assigned 60 min of homework per day had a standard deviation in time spent by students on homework of approximately 25 min, whereas in those schools assigning 120 min of homework, the standard deviation was twice as long, and was over 50 min. So schools which assigned more homework also tended to demonstrate greater differences in the time students need to spend on that homework.

**Figure 2:**
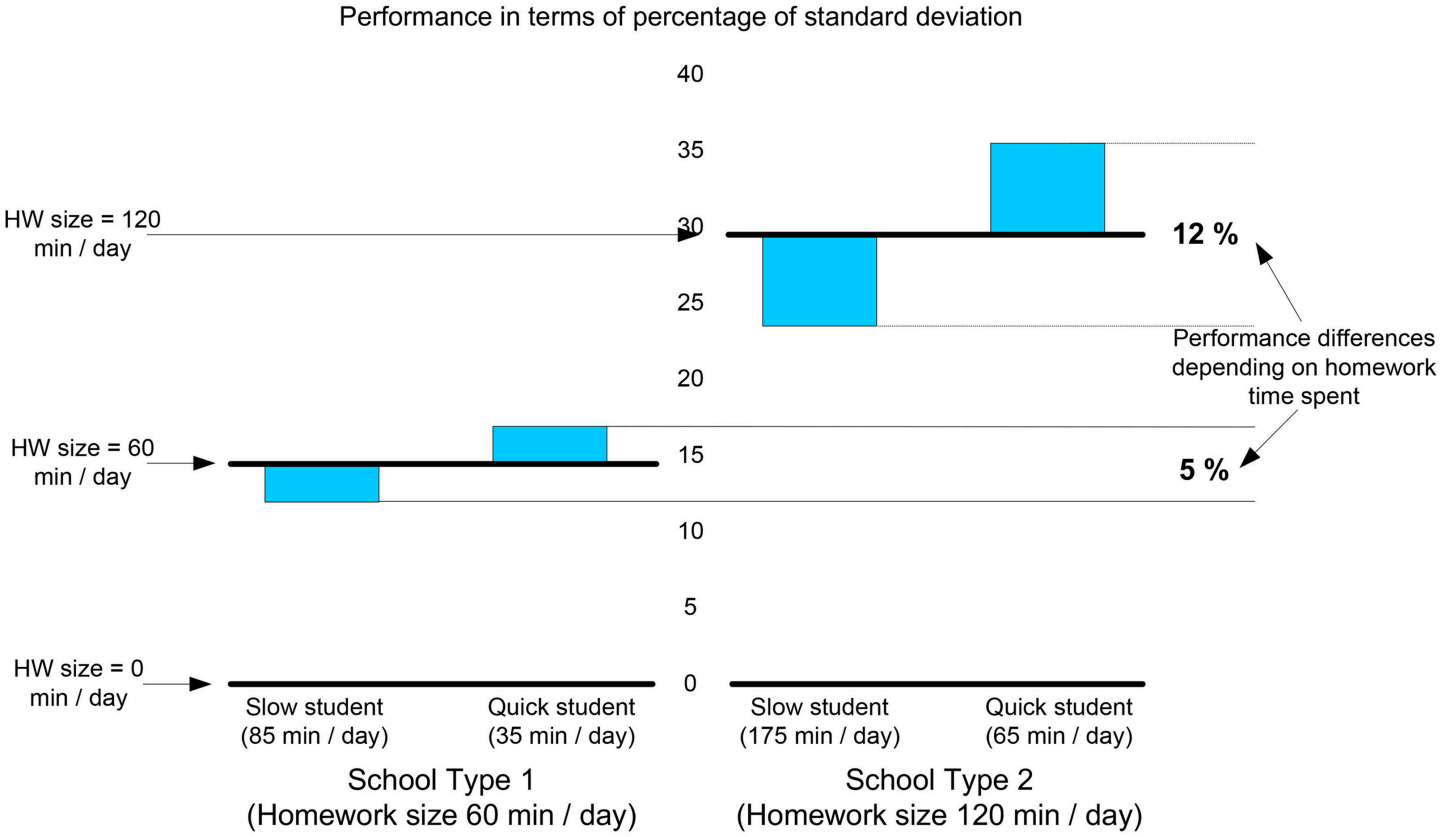
**Prediction of results for quick and slow students according to school homework size**.

Figure 2 shows the effect on results in mathematics of the combination of homework time, homework amount, and the variance of homework time associated with the amount of homework assigned in two types of schools: in type 1 schools the amount of homework assigned is 1 h, and in type 2 schools the amount of homework 2 h. The result in mathematics was used as a dependent variable because, as previously noted, it was the subject where the effect was smallest and as such is the most conservative prediction. With other subjects the results might be even clearer.

Looking at the first standard deviation of student homework time shown in the first graph, it was estimated that in type 1 schools, which assign 1 h of daily homework, a quick student (one who finishes their homework before 85% of their classmates) would spend a little over half an hour (35 min), whereas the slower student, who spends more time than 85% of classmates, would need almost an hour and a half of work each day (85 min). In type 2 schools, where the homework amount is 2 h a day, the differences increase from just over an hour (65 min for a quick student) to almost 3 h (175 min for a slow student). Figure 2 shows how the differences in performance would vary within a school between the more and lesser able students according to amount of homework assigned. In type 1 schools, with 1 h of homework per day, the difference in achievement between quick and slow students would be around 5% of a standard deviation, while in schools assigning 2 h per day the difference would be 12%. On the other hand, the slow student in a type 2 school would score 6 points more than the quick student in a type 1 school. However, to achieve this, the slow student in a type 2 school would need to spend five times as much time on homework in a week (20.4 weekly hours rather than 4.1). It seems like a lot of work for such a small gain.

## Discussion and conclusions

The data in this study reaffirm the multilevel nature of homework (Trautwein and Köller, 2003) and support this study's first hypothesis: the amount of homework (mean daily minutes the student spends on homework) is positively associated with academic results, whereas the time students spent on homework considered individually is negatively associated with academic results. These findings are in line with previous research, which indicate that school-level variables, such as amount of homework assigned, have more explanatory power than individual variables such as time spent (De Jong et al., 2000; Dettmers et al., 2010; Scheerens et al., 2013; Fernández-Alonso et al., 2015). In this case it was found that for each additional hour of homework assigned by a school, a gain of 25% of a standard deviation is expected in all subjects except mathematics, where the gain is around 15%. On the basis of this evidence, common sense would dictate the conclusion that frequent and abundant homework assignment may be one way to improve school efficiency.

However, as noted previously, the relationship between homework and achievement is paradoxical- appearances are deceptive and first conclusions are not always confirmed. Analysis demonstrates another two complementary pieces of data which, read together, raise questions about the previous conclusion. In the first place, time spent on homework at the individual level was found to have a negative effect on achievement, which confirms the findings of other multilevel-approach research (Trautwein, 2007; Trautwein et al., 2009b; Chang et al., 2014; Fernández-Alonso et al., 2016). Furthermore, it was found that an increase in assigned homework volume is associated with an increase in the differences in time students need to complete it. Taken together, the conclusion is that, schools with more homework tend to exhibit more variation in student achievement. These results seem to confirm our second hypothesis, as a positive covariation was found between the amount of homework in a school (the mean homework time by school) and the increase in differences within the school, both in student homework time and in the academic results themselves. The data seem to be in line with those who argue that homework is a source of inequity because it affects those less academically-advantaged students and students with greater limitations in their home environments (Kohn, 2006; Rømming, 2011; OECD, 2013b).

This new data has clear implications for educational action and school homework policies, especially in compulsory education. If quality compulsory education is that which offers the best results for the largest number (Barber and Mourshed, 2007; Mourshed et al., 2010), then assigning an excessive volume of homework at those school levels could accentuate differences, affecting students who are slower, have more gaps in their knowledge, or are less privileged, and can make them feel overwhelmed by the amount of homework assigned to them (Martinez, 2011; OECD, 2014b; Suárez et al., 2016). The data show that in a school with 60 min of assigned homework, a quick student will need just 4 h a week to finish their homework, whereas a slow student will spend 10 h a week, 2.5 times longer, with the additional aggravation of scoring one twentieth of a standard deviation below their quicker classmates. And in a school assigning 120 min of homework per day, a quick student will need 7.5 h per week whereas a slow student will have to triple this time (20 h per week) to achieve a result one eighth worse, that is, more time for a relatively worse result.

It might be argued that the differences are not very large, as between 1 and 2 h of assigned homework, the level of inequality increases 7% on a standardized scale. But this percentage increase has been estimated after statistically, or artificially, accounting for sociological and psychological student factors and other variables at school and region level. The adjustment variables influence both achievement and time spent on homework, so it is likely that in a real classroom situation the differences estimated here might be even larger. This is especially important in comprehensive education systems, like the Spanish (Eurydice, 2015), in which the classroom groups are extremely heterogeneous, with a variety of students in the same class in terms of ability, interest, and motivation, in which the aforementioned variables may operate more strongly.

The results of this research must be interpreted bearing in mind a number of limitations. The most significant limitation in the research design is the lack of a measure of previous achievement, whether an *ad hoc* test (Murillo and Martínez-Garrido, 2013) or school grades (Núñez et al., 2014), which would allow adjustment of the data. In an attempt to alleviate this, our research has placed special emphasis on the construction of variables which would work to exclude academic history from the model. The use of the repetition of school year variable was unavoidable because Spain has one of the highest levels of repetition in the European Union (Eurydice, 2011) and repeating students achieve worse academic results (Ministerio de Educación, 2011). Similarly, the expectation and motivation variables were included in the group of adjustment factors assuming that in this research they could be considered background variables. In this way, once the background factors are discounted, the homework variables explain 2% of the total variance, which is similar to estimations from other multilevel studies (De Jong et al., 2000; Trautwein, 2007; Dettmers et al., 2009; Fernández-Alonso et al., 2016). On the other hand, the statistical models used to analyze the data are correlational, and as such, one can only speak of an association between variables and not of directionality or causality in the analysis. As Trautwein and Lüdtke (2009) noted, the word “effect” must be understood as “predictive effect.” In other words, it is possible to say that the amount of homework is connected to performance; however, it is not possible to say in which direction the association runs. Another aspect to be borne in mind is that the homework time measures are generic -not segregated by subject- when it its understood that time spent and homework behavior are not consistent across all subjects (Trautwein et al., 2006; Trautwein and Lüdtke, 2007). Nonetheless, when the dependent variable is academic results it has been found that the relationship between homework time and achievement is relatively stable across all subjects (Lubbers et al., 2010; Chang et al., 2014) which leads us to believe that the results given here would have changed very little even if the homework-related variables had been separated by subject.

Future lines of research should be aimed toward the creation of comprehensive models which incorporate a holistic vision of homework. It must be recognized that not all of the time spent on homework by a student is time well spent (Valle et al., 2015). In addition, research has demonstrated the importance of other variables related to student behavior such as rate of completion, the homework environment, organization, and task management, autonomy, parenting styles, effort, and the use of study techniques (Zimmerman and Kitsantas, 2005; Xu, 2008, 2013; Kitsantas and Zimmerman, 2009; Kitsantas et al., 2011; Ramdass and Zimmerman, 2011; Bembenutty and White, 2013; Xu and Wu, 2013; Xu et al., 2014; Rosário et al., 2015a; Osorio and González-Cámara, 2016; Valle et al., 2016), as well as the role of expectation, value given to the task, and personality traits (Lubbers et al., 2010; Goetz et al., 2012; Pedrosa et al., 2016). Along the same lines, research has also indicated other important variables related to teacher homework policies, such as reasons for assignment, control and feedback, assignment characteristics, and the adaptation of tasks to the students' level of learning (Trautwein et al., 2009a; Dettmers et al., 2010; Patall et al., 2010; Buijs and Admiraal, 2013; Murillo and Martínez-Garrido, 2013; Rosário et al., 2015b). All of these should be considered in a comprehensive model of homework.

In short, the data seem to indicate that in year 8 of compulsory education, 60–70 min of homework a day is a recommendation that, slightly more optimistically than Cooper's (2001) “10 min rule,” gives a reasonable gain for the whole school, without exaggerating differences or harming students with greater learning difficulties or who work more slowly, and is in line with other available evidence (Fernández-Alonso et al., 2015). These results have significant implications when it comes to setting educational policy in schools, sending a clear message to head teachers, teachers and those responsible for education. The results of this research show that assigning large volumes of homework increases inequality between students in pursuit of minimal gains in achievement for those who least need it. Therefore, in terms of school efficiency, and with the aim of improving equity in schools it is recommended that educational policies be established which optimize all students' achievement.

## Ethics statement

This study was carried out in accordance with the recommendations of the University of Oviedo with written informed consent from all subjects. All subjects gave written informed consent in accordance with the Declaration of Helsinki. The protocol was approved by the University of Oviedo.

## Author contributions

RF and JM have designed the research; RF and JS have analyzed the data; MA and JM have interpreted the data; RF, MA, and JS have drafted the paper; JM has revised it critically; all authors have provided final approval of the version to be published and have ensured the accuracy and integrity of the work.

## Funding

This research was funded by the Ministerio de Economía y Competitividad del Gobierno de España. References: PSI2014-56114-P, BES2012-053488. We would like to express our utmost gratitude to the Ministerio de Educación Cultura y Deporte del Gobierno de España and to the Consejería de Educación y Cultura del Gobierno del Principado de Asturias, without whose collaboration this research would not have been possible.

### Conflict of interest statement

The authors declare that the research was conducted in the absence of any commercial or financial relationships that could be construed as a potential conflict of interest.
